# Food habits of 3 myrmecophilous bug species on myrmecophytic *Macaranga* (Malpighiales: Euphorbiaceae) vary from herbivory to predation

**DOI:** 10.1093/jisesa/iead078

**Published:** 2023-09-01

**Authors:** Usun Shimizu-kaya, Fujio Hyodo, Shouhei Ueda, Takashi Komatsu, Paulus Meleng, Takao Itioka

**Affiliations:** Faculty of Life and Environmental Sciences, Shimane University, 1060 Nishikawatsu-cho, Matsue, Shimane 690-8504, Japan; Research Core for Interdisciplinary Science, Okayama University, 3-1-1 Tsushima-naka, Okayama 700-8530, Japan; Department of Environmental Sciences and Technology, Graduate School of Agriculture, Osaka Metropolitan University, 1-1 Gakuen-cho, Naka-ku, Sakai, Osaka 599-8531, Japan; 2-4-3 Ninomiya, Tsukuba, Ibaraki 305-0051, Japan; Research and Development Division, Forest Department Sarawak, Kuching, Sarawak 93250, Malaysia; Graduate School of Human and Environmental Studies, Kyoto University, Yoshida-Nihonmatsu-cho, Sakyo-ku, Kyoto 606-8501, Japan

**Keywords:** ant defense, Hemiptera, Heteroptera, Lambir, ant–plant interactions

## Abstract

Myrmecophytes have mutualistic relationships with symbiotic ants. Although myrmecophytic *Macaranga* (Malpighiales: Euphorbiaceae) species are well protected by aggressive *Crematogaster* (Hymenoptera: Formicidae) ants, some bug species occur on the myrmecophytes. To clarify the associations of these bugs with the plants and the ants, we studied the food habits of 3 bug species, *Pilophorus lambirensis* Nakatani et Komatsu, 2013 (Hemiptera: Miridae: Phylinae), Phylinae sp. 1, and *Arbela* sp. 1 (Hemiptera: Nabidae). We conducted field observations in a Bornean rainforest. First, we located these bugs and studied their behavioral responses to the ants on *Macaranga* species; we then conducted stable isotope analyses. All bugs avoided direct contact with ants, but they occurred only on trees with active ants. *Pilophorus lambirensis* and Phylinae sp. 1 were most commonly observed on the apical parts of host trees, whereas *Arbela* sp. 1 was mainly in areas distant from the apical parts where ants were sparse. The stable isotope ratios indicated that Phylinae sp. 1 fed on food bodies, which are nutrient-rich spherical bodies produced by *Macaranga* trees on the apical parts for ants. Although the main diet of the other 2 species remains unclear, nitrogen isotopic signatures demonstrated that *P. lambirensis* is herbivorous, whereas *Arbela* sp. 1 is carnivorous. However, the distant location from ants and its isotopic signatures indicated that *Arbela* sp. 1 rarely fed on the ants. At least 2 mirid bug species might obtain enemy-free space in addition to the food provided by the myrmecophytes.

## Introduction

Myrmecophiles are organisms that live in association with ants for at least a certain period of their lives. Various taxa can be myrmecophiles, including both plants and arthropods (Hölldobler and Willson 1990). The associations between myrmecophiles and ants vary from mutualism to parasitism (Kronauer and Pierce 2011). In tropical regions, various ant species have mutualistic interactions with plants that have hollow spaces that can house ant colonies (Davidson and McKey 1993, Rico-Gray and Oliveira 2007). Plants that house ant colonies are called ‘myrmecophytes’, and the ants nesting in myrmecophytes are called ‘plant-ants’. Some arthropods, including ants, beetles, caterpillars, and spiders, can exploit ant–plant mutualistic interactions (Janzen 1975, Letourneau 1990, Meehan et al. 2009, Roux et al. 2011); they obtain nesting spaces, food, and enemy-free space.

The tree genus *Macaranga* (Malpighiales: Euphorbiaceae) contains more than 20 myrmecophytic species distributed in Southeast Asia (Fiala et al. 1999, Davies 2001). Most of the myrmecophytic *Macaranga* species have mutualistic associations with aggressive *Crematogaster* (Hymenoptera: Formicidae) species that inhabit their hollow stems (Fiala et al. 1999). The *Crematogaster* plant-ants intensively attack and remove intruders from host *Macaranga* trees (Fiala and Maschwitz 1992, Itioka et al. 2000). Despite being defended by ants, several lycaenid butterfly and phasmid species have been known to stay on *Macaranga* myrmecophytes and feed on the leaves (Maschwitz et al. 1984, Okubo et al. 2009, Shimizu-kaya and Itioka 2015). The chemical and physical defenses of myrmecophytes are often weaker than those of non-myrmecophytes because of a trade-off between ant defenses and non-ant (chemical and physical) defenses (Nomura et al. 2011). Thus, the leaves of *Macaranga* myrmecophytes are more palatable to herbivores than those of non-myrmecophytes.

Additionally, *Arhopala zylda* (Lepidoptera: Lycaenidae) larvae feed on food bodies of *Macaranga* myrmecophytes, which are nutrient-rich spherical products produced for plant-ants (Shimizu-kaya et al. 2014). These myrmecophilous insects on *Macaranga* myrmecophytes avoid plant-ant attacks using species-specific strategies, such as various combinations of chemical camouflage, honeydew production, and behavioral adaptations (Inui et al. 2015, Shimizu-kaya and Itioka 2015). Thus, *A. zylda* obtains palatable foods such as leaves and food bodies without interference by plant-ants.

At least 12 myrmecophilous bug species from 2 families have been documented on *Macaranga* myrmecophytes: 10 *Pilophorus* species (Hemiptera: Miridae: Phylinae), and 1 unidentified species each of Phylinae and *Arbela* (Hemiptera: Nabidae) (Nakatani et al. 2013, 2016, Shimizu-kaya et al. 2015). Because most *Pilophorus* species are polyphagous predators that feed on other small arthropods such as aphids and mites (Mansour et al. 2021), *Pilophorus* bugs might be beneficial to plants by reducing herbivore loads. However, *Pilophorus* spp. on *Macaranga* myrmecophytes might be parasites that prey on plant-ants on the myrmecophytes, where other small arthropods are rarely present (Itino and Itioka 2001). This might also be the case for *Arbela* species because most described Nabidae species are predators (Lattin 1989). To understand the roles of the myrmecophilous bugs in ant–plant mutualisms on *Macaranga* myrmecophytes, their food habits require clarification.

In contrast to the above prediction, *Pilophorus* spp. on *Macaranga* myrmecophytes did not prey on ants that were artificially weakened under laboratory-rearing conditions (T.K., unpublished data). However, *Pilophorus lambirensis* Nakatani et Komatsu, 2013 and a few other *Pilophorus* species frequently fed on food bodies under laboratory-rearing conditions in the absence of plant-ants (Nakatani et al. 2013, 2016, Shimizu-kaya et al. 2015). It is reasonable to infer that the *Pilophorus* and the unidentified Phylinae species (Phylinae sp. 1) are potentially capable of using food bodies on *Macaranga* myrmecophytes as their main food source because *Macaranga* food bodies are rich in nitrogen (Heil et al. 1998, Hatada et al. 2002); many mirid bug species prefer to feed on tissues rich in nitrogen (Wheeler 2001). However, it remains unclear whether these insects engage in these food habits under field conditions in the presence of aggressive plant-ants.

Two nymphs of the unidentified *Arbela* species (*Arbela* sp. 1) fed on plant-ants under laboratory-rearing conditions (personal observation). However, previous observations of *Arbela* sp. 1 have shown that they are present on the mature parts of the host trees where plant-ants are sparse, and they feed on food bodies under laboratory-rearing conditions in the absence of plant-ants (Shimizu-kaya et al. 2015). Therefore, the food habits of *Arbela* sp. 1 in the field also remain uncertain.

In this study, we focused on the food habits of the 3 myrmecophilous bug species, *P. lambirensis*, Phylinae sp. 1, and *Arbela* sp. 1, on 3 *Macaranga* myrmecophyte species in a Bornean tropical rainforest. We predicted that 2 mirid bug species, *P. lambirensis* and Phylinae sp. 1, feed on food bodies, whereas *Arbela* sp. 1 mainly feeds on plant-ants and occasionally on food bodies. To test these predictions, we surveyed for the presence of plant-ants on plants on which the bugs also occurred and determined the site preferences of bugs within a tree and the behavioral responses of bugs to plant-ants. Moreover, we measured the nitrogen (N) and carbon (C) stable isotope ratios of the myrmecophilous bugs and their potential food resources. As the general patterns in the food web for stable isotope ratios, δ^15^N values of consumers feeding on invertebrates and plants are 1.4 ± 0.2‰ and 2.2 ± 0.3‰ higher than those of their food resources, respectively, whereas their δ^13^C values increase from those of their food resources by only approximately 1‰ (McCutchan et al. 2003). Stable isotope analyses have been used to investigate the nutritional interactions of ants and other invertebrates on myrmecophytes (e.g., Tillberg 2004, Meehan et al. 2009, McNett et al. 2010).

## Materials and Methods

### Study Site

This study was conducted in Lambir Hills National Park, Miri, Sarawak, Malaysia (4°11ʹN, 114°02ʹE; altitude, 150–250 m) in Borneo from 2012 to 2013. The area is covered with lowland mixed dipterocarp forest (Inoue and Hamid 1994, Yumoto and Nakashizuka 2005). *Macaranga* plants commonly grow in forest gaps, along riversides, and at forest edges (Davies 1998). There are no clear dry seasons in a year (Kume et al. 2011). Mean annual temperature and mean annual rainfall at the site were approximately 27 °C and 2,600 mm, respectively (Kumagai et al. 2009).

### Bugs

**Fig. 1.**
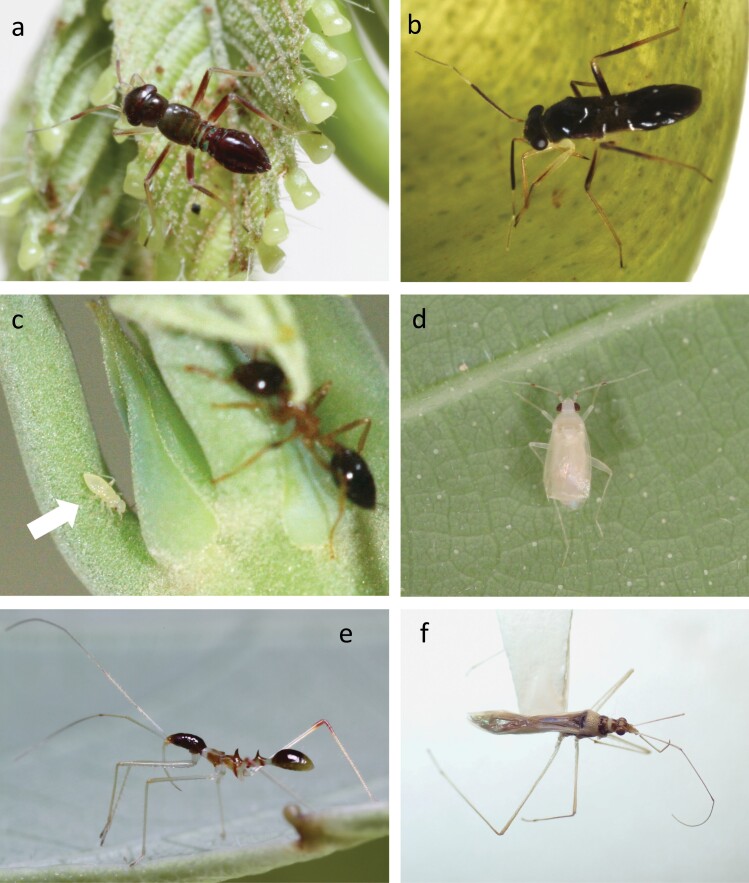
The 3 myrmecophilous bug species examined in the study. *Pilophorus lambirensis*: nymph a) and adult b); Phylinae sp. 1: nymph c) and adult d); and *Arbela* sp. 1: nymph e) and adult f).

**Fig. 2.**
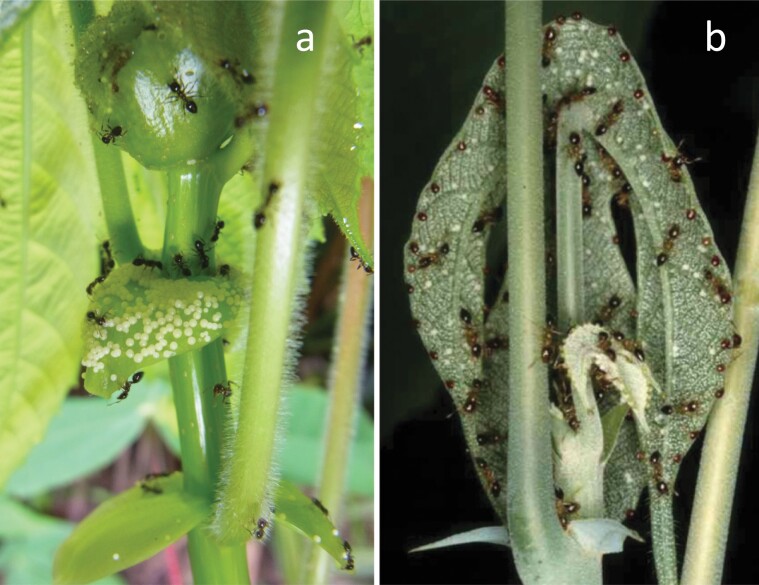
Food bodies attended by plant-ants on young stipules of *Macaranga hosei* a) and young leaves of *M. beccariana* b).

At least 9 *Pilophorus* species inhabit myrmecophytic *Macaranga* plants at the study site (Nakatani et al. 2013, 2016). In this study, we focused on *P. lambirensis*, which occurred more frequently on the host plants than other *Pilophorus* species (personal observation). They are small castaneous bugs that reach 3.16 mm in body length as adults (Nakatani et al. 2013, Fig. 1a and b), and inhabit *M. hosei* and *M. rufescens* (Nakatani et al. 2013). An unidentified phyline species, Phylinae sp. 1, has a stark white body that is 1.7 mm in length and only occurs on *M. beccariana* (Shimizu-kaya et al. 2015, personal observation, Fig. 1c and d). *Arbela* sp. 1 inhabits *M. beccariana* (Shimizu-kaya et al. 2015). Their body length reaches 5.5 mm (personal observation, Fig. 1e and f). *Pilophorus lambirensis* and *Arbela* sp. 1 morphologically resemble the plant-ants (Fig. 2) on *Macaranga* species in body coloration, particularly in their nymphal stages (personal observation). Specimens of the 3 bug species were deposited in the Kuching Insectarium of Forest Department Sarawak.

### *Macaranga* Myrmecophytes and Their Plant-Ants

Three *Macaranga* species, *M. hosei*, *M. rufescens*, and *M. beccariana*, are host plants of the bug species examined in this study. They are obligate myrmecophytic species that have hollow stems and peltate leaves with long petioles (Fiala et al. 1994, Itioka 2005). *Macaranga hosei* and *M. rufescens* are closely related (Davies 2001) and are mainly associated with *Crematogaster borneensis* and *C. linsenmairi*, whereas *M. beccariana* is associated with *C. decamera* (Fiala et al. 1999, Feldhaar et al. 2016).

On these *Macaranga* myrmecophytes, a symbiotic association starts with colony foundation by a plant-ant queen inside the hollow stem of a *Macaranga* seedling, when the seedling reaches approximately 70–100 cm in height for *M. hosei* and *M. rufescens*, or approximately 10 cm in height for *M. beccariana* (Fiala and Maschwitz 1992). Around the same time, the seedling starts producing food bodies on the adaxial surface of the orbicular, flap-like stipules on younger stems for *M. hosei* and *M. rufescens*, or the abaxial surface of the youngest apical leaves for *M. beccariana* (Fiala and Maschwitz 1992, Davies 2001; Fig. 2). Hereafter, we used “ants” to refer to “plant-ants”.

Ants on *Macaranga* myrmecophytes depend on food bodies as their main food source and behave as herbivores (Fiala et al. 1989, Hashimoto et al. 1997, Itino et al. 2001, Hyodo et al. 2011). They continuously patrol the plant surface to clean extraneous substances, collect food bodies and extrafloral nectar (EFN) secreted on the leaves, and protect their host plants from herbivores on the plant surface by purging them with their aggressive behavior and mass recruiting system (Fiala et al. 1989, 1991; Itioka et al. 2000). They preferentially protect young parts of their host plants (Itioka et al. 2000, Heil et al. 2004). Thus, food bodies and extrafloral nectaries are usually continuously attended by ants on the 3 *Macaranga* species. However, ant activity on the plant surface and ant abundance on an individual host tree have been occasionally observed to decrease following disturbances of the ant colony, such as the destruction of stems by ant-feeding woodpeckers and ant predation by lizards and spiders (Shimizu-kaya et al. 2015). The production of food bodies and EFN on *Macaranga* myrmecophytes is reduced when ants are absent (Heil et al. 1997).

### Field Observations

We observed 267 trees of the targeted *Macaranga* species (32, 17, and 218 of *M. hosei*, *M. rufescens*, and *M. beccariana*, respectively), the height of which ranged between 0.3 and 6.0 m, and checked for the presence of the 3 bug species in the daytime. The upper parts of taller trees were checked from nearby slopes or the trunks of fallen trees. When no ants were found on the surface of a tree, we assumed that the ants were not actively defending the tree. Among the trees surveyed, active ant defenses were absent on 9 *M. hosei*, 1 *M. rufescens*, and 10 *M. beccariana* trees.

We categorized the above-ground part of a tree into 2 sections: food body-bearing and non-food body-bearing. The border between the 2 sections on each branch was at the basal node of the food body-bearing leaf (in *M. beccariana*) or food body-bearing stipules (in *M. hosei* and *M. rufescens*) closest to the roots; the food body-bearing section on each branch comprised the stem, stipule, and leaf parts at the apical side from the border, and the non-food body-bearing section on the branch comprised of the other parts, ranging from the border to the base.

When we found any individuals of the bug species on a targeted tree, we recorded the locations of the bug individuals within the tree and then observed the behavior of each bug individual at least 10 min. We observed 9 adults and 15 nymphs of *P. lambirensis* on 7 *M. hosei* trees, and 18 adults and 28 nymphs of *P. lambirensis* on 10 *M. rufescens* trees. We observed 10 adults and 9 nymphs of Phylinae sp. 1 on 6 *M. beccariana* trees, and 17 adults and 15 nymphs of *Arbela* sp. 1 on 16 *M. beccariana* trees.

### Stable N and C Isotope Analysis

We conducted stable N and C isotope analyses to examine whether the myrmecophilous bugs feed on either ants or food bodies in the field. For this analysis, we collected 2 and 5 *P. lambirensis* adults from 2 *M. hosei* and 3 *M. rufescens* trees, respectively; 19 Phylinae sp. 1 adults from 5 *M. beccariana* trees; and 13 adult *Arbela* sp. 1 from 7 *M. beccariana* trees. We also collected mature leaves, food bodies, and ants from each of the trees from which bugs were collected. The samples were oven-dried at 60 °C for 48 h in the laboratory at the study site. The arthropods were kept in a freezer in the laboratory for 3 h to euthanize them prior to oven drying.

The whole bodies of the arthropods and plant materials were ground into powder using a mortar and pestle, and a mixer, respectively. Bug individuals of the same species collected from the same tree were combined into 1 sample (Supplementary Tables S1 and S2). A few ant individuals per tree were combined into 1 sample. The samples were placed in tin capsules, and the isotope ratios were measured using a mass spectrometer (Delta ^plus^ XP, Bremen, Germany) coupled with an elemental analyzer. The natural abundances of ^15^N and ^13^C are expressed in per mil (‰) deviation from international standards: δ^15^N or δ^13^C = (R_sample_/R_standard_ − 1) × 1,000, where R in δ^15^N or δ^13^C is ^15^N/^14^N or ^13^C/^12^C, respectively. Atmospheric N and Pee Dee belemnite were used as the international standards for N and C, respectively. The analytical precision was better than 0.2‰ for both N and C isotopes.

### Statistical Analysis

Binomial tests were performed to examine whether the number of bugs in the 2 within-tree sections (food body-bearing and non-food body-bearing) was equal for each bug and plant species. These analyses were conducted using R ver. 4.0.2 (R Core Team 2020). We did not statistically compare the presence of bugs on trees with actively defended by ants and not actively defended by ants because of the small sample size of the latter for each *Macaranga* species.

We used linear mixed models to test whether δ^15^N or δ^13^C values differed among the following categories: *P. lambirensi*s, ants, food bodies, and leaves on *M. hosei* and *M. rufescens*; and Phylinae sp. 1 and *Arbela* sp. 1, ants, food bodies, and leaves on *M. beccariana.* In the linear mixed models, the δ^15^N or δ^13^C values were the response variables, the categories were the explanatory variables, and the identity of the host tree was treated as a random effect. The *M. hosei* and *M. rufescens* samples were pooled because of the small sample size of the 2 *Macaranga* species, the very close phylogenetic relationship between the 2 species, and the similar isotopic patterns of the samples for each category (Supplementary Tables S1 and S2). The 2 *Macaranga* species were also included as a random effect. Tukey–Kramer HSD post hoc tests were used to test for differences among the categories. The analyses were performed in JMP ver. 13 (SAS Institute, Cary, NC, USA).

## Results

### Field Observations

*Pilophorus lambirensis* were observed on 8 *M. hosei* and 10 *M. rufescens* trees (Table 1). All the trees where *P. lambirensis* were present had colonies of their specific ant species and were actively defended by ants on the plant surfaces. Among the 10 trees of *M. hosei* and *M. rufescens* that were not actively defended by ants, 7 were too young and did not yet house ants.

**Table 1.** Number of *Macaranga* trees on which at least one individual of the 3 bug species, *Pilophorus lambirensis*, Phylinae sp. 1, and *Arbela* sp. 1, was observed (‘with bugs’), and trees on which no bugs of the 3 species were observed (‘without bugs’). Trees were considered to be not actively defended by ants (i.e., inactive) when no plant-ants were observed on the surface of the trees

*Macaranga*	Ant defense	With bugs				Without bugs	Total
		Pl	Ph	Ar	Ph+Ar		
*hosei*	Active	8	0	0	0	15	23
	Inactive	0	0	0	0	9	9
*rufescens*	Active	10	0	0	0	6	16
	Inactive	0	0	0	0	1	1
*beccariana*	Active	0	8	15	4	181	208
	Inactive	0	0	0	0	10	10

Pl, *Pilophorus lambirensis*; Ph, Phylinae sp. 1; Ar, *Arbela* sp. 1.

All *P*. *lambirensis* individuals were observed on food body-bearing sections on both *M. hosei* and *M. rufescens* (Table 2), and they were present on the abaxial and adaxial surfaces of young leaves. Two adults and 3 nymphs on *M. hosei*, and 1 adult on *M. rufescens* were observed moving from a young leaf to another young leaf or stipules in response to being close to patrolling ants, which allowed them to maintain their distance from other ants. In general, direct contact was not observed between *P. lambirensis* and ants on *M. hosei* and *M. rufescens*; however, in one instance, several ants surrounded, bit, and finally killed an adult *P. lambirensis* on the abaxial side of a young leaf of *M. rufescens*. We did not observe any feeding behaviors of *P. lambirensis* on the plant surface.

**Table 2.** Total number of nymphs and adults of the 3 bug species on each section within a *Macaranga* tree.

Bug	Within-tree section		Binomial test
	FB-bearing	Non-FB-bearing	
*Pilophorus lambirensis* on *M. hosei*	27	0	*P* < 0.0001
*P. lambirensis* on *M. rufescens*	46	0	*P* < 0.0001
Phylinae sp. 1 on *M. beccariana*	24	0	*P* < 0.0001
*Arbela* sp. 1 on *M. beccariana*	4	28	*P* < 0.0001

FB, food body.

Phylinae sp. 1 and *Arbela* sp. 1 were found on 12 and 19 trees, respectively, of the surveyed *M. beccariana* trees (Table 1). Of those *M. beccariana* trees, 4 trees had both bug species. All the trees that harbored the 2 bug species had a colony of ants that actively defended the plant surfaces.

All the observed adults and nymphs of Phylinae sp. 1 were present on the food body-bearing section of their host plant (Table 2). They were found on the abaxial side of young apical leaves and often gathered around the petiole insertion of the leaves. One adult Phylinae sp. 1 was observed to move to a nearby stipule in response to approaching ants, and they returned to the leaves within 5 min. For Phylinae sp. 1, no direct contact with ants and feeding behavior were observed on the plant surface.

*Arbela* sp. 1 was present on non-food body-bearing sections more frequently than on food body-bearing sections (Table 2). On non-food body-bearing sections, they were present on the abaxial and adaxial surfaces of the leaves. For *Arbela* sp. 1, no direct contact with ants and feeding behavior were observed on the plant surface. We did observe an adult flying from a mature leaf to another mature leaf of the same tree, although no ants had approached them.

### Stable N and C Isotope Analysis

#### *Pilophorus lambirensis* on *M. hosei* and *M. rufescens*

**Fig. 3.**
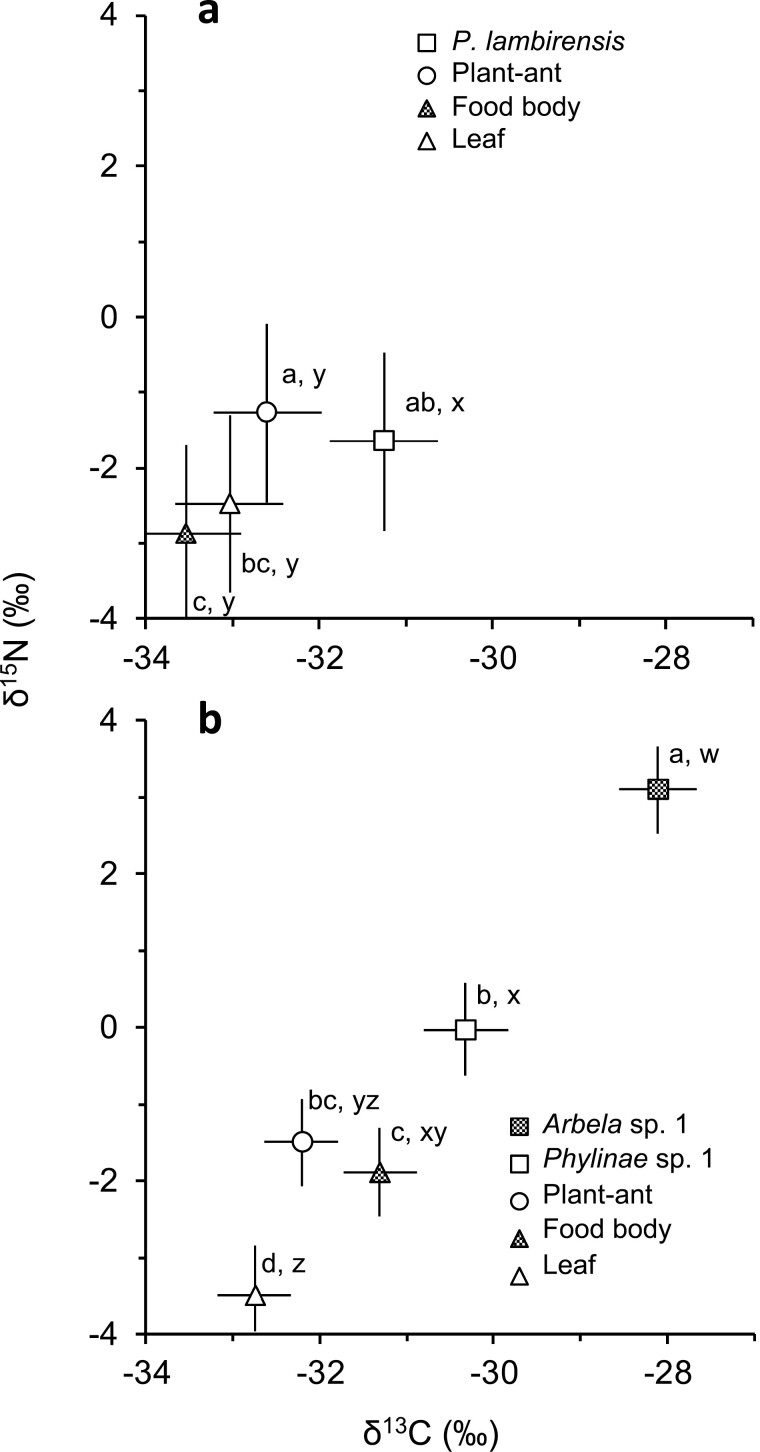
δ^15^N and δ^13^C values (least squares mean ± SE) for a) *Pilophorus lambirensis* on *Macaranga hosei* and *M. rufescens* and for b) Phylinae sp. 1 and *Arbela* sp. 1 on *M. beccariana* relative to plant-ants, food bodies, and leaves of their host. a, b, c, and d represent significant differences at *P* < 0.05 (Tukey–Kramer HSD post hoc test) for δ^15^N, whereas w, x, y, and z represent significant differences for δ^13^C.

The δ^15^N and δ^13^C values significantly differed among the bugs, ants, food bodies, and leaves of their host trees (δ^15^N: *F*_3, 12_ = 11.71, *P* = 0.0007; δ^13^C: *F*_3, 12_ = 11.22, *P* = 0.0008; Fig. 3a, Supplementary Tables S1, S2). *Pilophorus lambirensis* showed significantly higher δ^13^C than the other categories (least squares mean ± SE: −31.3 ± 0.6‰ for *P. lambirensis*, −32.6 ± 0.6‰ for ants, −33.5 ± 0.6‰ for food bodies, −33.0 ± 0.6‰ for leaves). The δ^15^N of *P. lambirensis* was significantly higher than that of food bodies (−1.7 ± 1.2‰ for *P. lambirensis*, −2.9 ± 1.2‰ for food bodies). The δ^15^N of *P. lambirensis* was intermediate between the ant and leaf values.

#### Phylinae sp. 1 and *Arbela* sp. 1 on *M. beccariana*

The δ^15^N and δ^13^C values significantly differed among the 2 bug species, ants, food bodies, and leaves of *M. beccariana* (δ^15^N: *F*_4, 26.2_ = 56.39, *P* < 0.0001; δ^13^C: *F*_4, 26.2_ = 49.94, *P* < 0.0001; Fig. 3b, Supplementary Tables S1, S2). *Arbela* sp. 1 had significantly higher δ^15^N (3.1 ± 0.6‰) and δ^13^C (−28.1 ± 0.4‰) than the other categories. The δ^15^N and δ^13^C values of Phylinae sp. 1 were lower than those of *Arbela* sp. 1 by approximately 3.1‰ and 2.2‰, respectively. Phylinae sp. 1 had significantly higher δ^15^N than food bodies and leaves (−0.0 ± 0.6‰ for Phylinae sp. 1, −1.9 ± 0.6‰ for food bodies, −3.5 ± 0.6‰ for leaves). The δ^15^N value of Phylinae sp. 1 was higher than that of ants by approximately 1.5‰, but the differences were not significant. However, Phylinae sp. 1 had significantly higher δ^13^C than ants and leaves (−30.3 ± 0.5‰ for Phylinae sp. 1, −32.2 ± 0.4‰ for ants, −32.8 ± 0.4‰ for leaves). The δ^13^C value of food bodies (−31.3 ± 0.4‰) was not significantly different from that of Phylinae sp. 1.

## Discussion

Our field observations demonstrated that all individuals of the 3 bug species observed in this study inhabited the trees that ants actively defended. Although we could not detect any feeding behaviors during our field observations for the 3 myrmecophilous bug species, the results of stable isotope analysis and behavioral traits, such as the position within the tree of each bug species, gave us clues about their food habits in the field.

The similarity in δ^15^N values between *P. lambirensis* and ants on *M. hosei* and *M. rufescens* indicated that the bugs likely did not prey on ants and other herbivores of the host trees, and that the bugs probably fed on plant materials. Contrary to our prediction, the δ^13^C values of *P. lambirensis* were 2.3‰ higher than those of food bodies, which indicated that the bugs did not feed solely on food bodies. The observed behaviors of *P. lambirensis* in response to approaching ants revealed that ants likely prevent *P. lambirensis* from accessing the nutrient-rich food bodies. Other probable food sources for *P. lambirensis* were the flesh of young leaves and EFN secreted on the glands along the margins of young leaves, which was their preferred site. These materials are known to be consumed by some herbivorous and omnivorous mirid bug species (Wheeler 2001, Sharma et al. 2014). The higher δ^13^C values of *P. lambirensis* compared with those of mature leaves provides support for this possibility given that young leaves are depleted in ^13^C relative to mature leaves (Cernusak et al., 2009), and nectars tend to be depleted in ^13^C relative to leaves (Lüttge et al. 1985).

The lack of significant difference in δ^15^N between Phylinae sp. 1 and the ants indicates that they are at similar trophic levels. Considering that the mean δ^13^C values of Phylinae sp. 1 were similar to that of food bodies, food bodies are likely a food source for this bug species. However, Phylinae sp. 1 occasionally showed somewhat higher δ^13^C values than food bodies (up to 2.7‰) and higher δ^15^N values than ants (up to 3.4‰) (Supplementary Tables S1 and S2). This suggests that Phylinae sp. 1 might be an omnivore that sometimes preys on ants or other animals, most of which are at the same trophic levels as ants, in addition to food bodies.

Regardless of the variation in the dependency on food bodies as food source among Phylinae sp. 1, food body feeding was consistent with our observation that this bug species preferred the abaxial side of food body-bearing young leaves as their microhabitats within a host tree. Similar to *P. lambirensis* on *M. hosei* and *M. rufescens*, Phylinae sp. 1 seems to suffer interference in access to food bodies from ants, which were observed to continuously attend food body-bearing young leaves on *M. beccariana*. However, Phylinae sp. 1 may be able to evade interference by ants through chemical camouflage, for example, as has been observed in coreid bugs on myrmecophytic acacia plants (Coronado-Rivera et al. 2020). Additionally, differences in food body-bearing sites between host species can lead to differences in accessibility to food bodies between the 2 bug species; food bodies are produced on the young leaves in *M. beccariana* and on the stipules in *M. hosei* and *M. rufescens* (Fig. 2).

The results of stable N isotope analysis indicate that *Arbela* sp. 1 is carnivorous; this is consistent with what is known about the other members of Nabidae, which are also carnivorous (Lattin 1989). However, the δ^13^C values of the species were unexpectedly high compared with those of Phylinae sp. 1 and ants on *M. beccariana* (Fig. 3b). In addition, *Arbela* sp. 1 was more frequently observed on the non-food body-bearing sections (Table 1), which are less intensively and less frequently attended by ants. These results suggest that *Arbela* sp. 1 is a generalist predator that mostly feeds on various types of prey on *M. beccariana*, even though they may opportunistically feed on ants or food bodies, as observed under laboratory-rearing conditions (Shimizu-kaya et al. 2015).

According to a previous study showing that the production of food bodies and EFN is low on *Macaranga* myrmecophytes when ants are absent (Heil et al. 1997), the food resources of the 2 mirid bug species, Phylinae sp. 1 and *P. lambirensis*, are more abundant on trees that ants actively defend rather than trees that are not actively defended by ants. Moreover, the latter of each studied *Macaranga* species was frequently infested by leaf-chewing herbivores, such as the larvae of *Aetholix flavibasalis* (Guenée, 1854) (Lepidoptera, Crambidae) that feed on the shoots, young leaves, and food bodies, during our field survey (*per. obs.*). Itioka et al. (2000) demonstrated that myrmecophytic *Macaranga* trees experience much damage from herbivores when they are no longer defended by ants. Therefore, the 2 mirid bug species would occur on the trees on which aggressive ants are present to obtain abundant food without interference from other herbivores on the host trees, although they should avoid contact with ants.

Aside from access to food resources, 1 plausible benefit of inhabiting *Macaranga* myrmecophytes with aggressive ants is to escape from their natural enemies (i.e., obtain enemy-free space), as has been reported in other myrmecophilous bug species (Maschwitz and Klinger 1974, Maschwitz et al. 1987, Gibernau and Dejean 2001). The presence of ants might decrease the rate of predation and parasite pressure on the bugs, especially on *P. lambirensis* and Phylinae sp. 1, which inhabit the young parts of host trees with abundant ants. The myrmecomorphy and spatial proximity to ants in *Pilophorus* spp. might indicate that they are Batesian mimic of ants (Yasunaga and Schuh 2013). This would also apply to *P. lambirensis*, which is morphologically similar to ants.

In contrast to the 2 mirid bug species, *Arbela* sp. 1 was present on the mature parts of the host plant and did appears to feed on plant materials. It remains unclear whether the benefits of obtaining food resources and enemy-free space also apply to *Arbela* sp. 1. However, no *Arbela* sp. 1 individuals were found on the other 11 species of *Macaranga* myrmecophytes distributed in the study site and the nymphs morphologically mimicked *C. decamera* ants, which are associated with *M. beccariana* (Shimizu-kaya et al. 2015); therefore, *Arbela* sp. 1 appears to be adapted to living on *M. beccariana*. This bug species may act as an additional anti-herbivore defense for host myrmecophytes, as seen in *Zelus annulosus* (Hemiptera: Reduviidae), which is a myrmecophile of *Allomerus* ants associated with myrmecophytic *Hirtella* plants in French Guiana and preys on their herbivores (Revel et al. 2010).

Overall, the results of this study revealed major differences in the food habits of the 3 myrmecophilous bug species on *Macaranga* myrmecophytes, as well as the 2 mirid bug species. The mirid bugs indicate that myrmecophytic *Macaranga* trees could provide palatable, nutrient-rich food resources and enemy-free space as long as the myrmecophilous organisms adapt to life with aggressive ants. Although the 3 bug species engaged in similar behavior to evade the ants, they also employed different strategies to prevent interference from ants. Experiments in which each of these bug species is introduced into non-host *Macaranga* species or trees attended by ant species or colonies that are different from the ones on their original host are needed to reveal the strategies used by these bug species to thwart ant attack.

## Supplementary Material

iead078_suppl_Supplementary_Information
