# Supplementary material for: Food habits of 3 myrmecophilous bug species on myrmecophytic Macaranga (Malpighiales: Euphorbiaceae) vary from herbivory to predation
Source: J Insect Sci. 2023 Sep 1;23(5):2. doi: 10.1093/jisesa/iead078 (PMC10473451; doi:10.1093/jisesa/iead078)
Supplement: iead078_suppl_Supplementary_Information [file iead078_suppl_supplementary_information.docx]

**SUPPORTING INFORMATION**

**Table S1.** δ^15^N values (‰) of each sample of *Pilophorus lambirensis* on *Macaranga hosei* and *M. rufescens*, Phylinae sp. 1 and *Arbela* sp. 1 on *M. beccariana*, and plant-ants, food bodies, and leaves of the trees on which the bugs were collected. The numbers in parentheses indicate the number of bug individual(s) treated as one sample.

**-------------------------------------------------------------------------------------------------**

Tree No. Pl Ph Ar ants FB leaves

-------------------------------------------------------------------------------------------------

*Macaranga hosei*

1 −1.3 (2) −1.1 −2.4 −2.1

2 −4.1 (2) −4.4 −5.9 −5.1

*M. rufescens*

1 −0.5 (1) 0.9 −2.1 −0.6

2 0.0 (1) −0.1 −0.4 −1.1

3 −1.6 (3) −0.9 −2.8 −2.7

*M. beccariana*

1 2.6 (1) 0.6 0.3 −0.9

2 1.3 (2) 0.9 0.2 −1.0

3 0.8 (7) 3.1 (1) −0.6 −1.9 −2.3

4 4.2 (2) 0.7 −0.3 −3.0

5 −0.2 (2) −1.3 −2.3 −3.1

6 2.4 (6) −1.8 −2.2 −4.7

7 −0.7 (3) 2.0 (1) −4.0 −3.7 −5.4

8 −2.0 (5) 2.5 (1) −4.0 −3.7 −5.4

9 3.0 (1) −4.0 −3.4 −7.1

**--------------------------------------------------------------------------------------------------**

Abbreviations: *Pilophorus lambirensis* (Pl), Phylinae sp. 1 (Ph), *Arbela* sp. 1 (Ar), plant-ants (ants), food bodies (FB) **Table S2.** δ^13^C values (‰) of each sample of *Pilophorus lambirensis* on *Macaranga hosei* and *M. rufescens*, Phylinae sp. 1 and *Arbela* sp. 1 on *M. beccariana*, and plant-ants, food bodies, and leaves of the trees on which the bugs were collected. The numbers in parentheses indicate the number of bug individual(s) treated as one sample.

**----------------------------------------------------------------------------------------------------**

Tree No. Pl Ph Ar ants FB leaves

----------------------------------------------------------------------------------------------------

*Macaranga hosei*

1 −29.6 (2) −30.8 −32.9 −32.8

2 −31.6 (2) −31.5 −33.7 −32.9

*M. rufescens*

1 −33.9 (1) −35.2 −35.2 −34.6

2 −30.4 (1) −32.5 −33.0 −32.6

3 −30.5 (3) −32.7 −32.6 −32.0

*M. beccariana*

1 −28.6 (1) −33.1 −32.6 −33.9

2 −33.0 (2) −33.8 −32.8 −34.2

3 −29.5 (7) −29.0 (1) −32.5 −30.6 −32.5

4 −27.1 (2) −32.5 −31.0 −33.3

5 −30.6 (2) −33.0 −32.9 −33.1

6 −28.1 (6) −31.1 −30.5 −32.1

7 −30.2 (3) −27.6 (1) −32.8 −32.9 −33.5

8 −29.4 (5) −26.8 (1) −30.4 −29.2 −30.7

9 −27.3 (1) −30.7 −29.3 −32.5

**-----------------------------------------------------------------------------------------------------**

Abbreviations: *Pilophorus lambirensis* (Pl), Phylinae sp. 1 (Ph), *Arbela* sp. 1 (Ar), plant-ants (ants), food bodies (FB)
